# Integrated multi-omics analysis for lung adenocarcinoma in Xuanwei, China

**DOI:** 10.18632/aging.205300

**Published:** 2023-12-13

**Authors:** Boyi Jiang, Jiapeng Yang, Rui He, Dong Wang, Yunchao Huang, Guangqiang Zhao, Mingjie Ning, Teng Zeng, Guangjian Li

**Affiliations:** 1 Department of Thoracic Surgery, The Third Affiliated Hospital of Kunming Medical University, Yunnan Cancer Hospital, Kunming, Yunnan 650032, China

**Keywords:** lung adenocarcinoma, Xuanwei lung cancer, oxidative phosphorylation, VIPR1, redox metabolism

## Abstract

Background: Xuanwei lung cancer (XWLC) is well-known for its high incidence and mortality. However, the molecular mechanism is still unclear.

Methods: We performed a comprehensive transcriptomic, proteomic, and phosphoproteomic characterization of tumors and matched normal adjacent tissues from three XWLC patients with lung adenocarcinoma (LUAD).

Results: Integrated transcriptome and proteome analysis revealed dysregulated molecules and pathways in tumors and identified enhanced metabolic-disease coupling. Non-coding RNAs were widely involved in post-transcriptional regulatory mechanisms to coordinate the progress of LUAD and partially explained the molecular differences between RNA and protein expression patterns. Phosphoproteome provided evidence support for new phosphate sites, reporting the potential roles of core kinase family members and key kinase pathways involved in metabolism, immunity, and homeostasis. In addition, by comparing with the previous LUAD researches, we emphasized the higher degree of oxidative phosphorylation in Xuanwei LUAD and pointed that VIPR1 deficiency aggravated metabolic dysfunction.

Conclusion: Our integrated multi-omics analysis provided a powerful resource for a systematic understanding of the molecular structure of XWLC and proposed therapeutic opportunities based on redox metabolism.

## INTRODUCTION

Lung cancer is the leading cause of cancer related mortality in China. Although smoking is the main factor in the development of lung cancer, the high incidence of lung cancer among non-smokers is related to genetic factors and environmental carcinogen exposure [1]. Xuanwei city, a rural area in southwestern China (Yunnan Province) with the highest incidence and mortality rates of lung cancer, is mainly caused by the burning of smoked coal without indoor ventilation [2–4]. Although indoor air pollution in Xuanwei has been well controlled after the 1980s, the higher incidence and mortality of lung cancer have not changed significantly [5]. This evidence indicates that the mechanism of Xuanwei lung cancer (XWLC) has not been well understood and should be further investigated. Moreover, most previous studies focused on the epidemiology and etiology of XWLC, but the understanding of the molecular basis of XWLC is still insufficient.

The tumorigenesis and development of lung cancer is a complicated pathological process, which involves multi-gene participation and multi-level development. A complex molecular network interaction accompanied by the abnormal expression of several genes is formed to jointly regulate the biological process of tumor cells, resulting in tumor invasion and metastasis [6]. Although some progress has been made in XWLC chemotherapy and targeted molecular therapy in recent years, but the overall 5-year survival rate is still less than 15% due to the limitations of treatment options, tumor metastasis, and recurrence [7]. Therefore, any single study is unlikely to elucidate the molecular mechanism of human lung cancer. Multi-omics methods provide a powerful tool for comprehensively and systematically revealing tumor progression pathways and essential biomarkers. Recently, the genomic, transcriptomic, proteomic, and epigenomics of the large-scale lung adenocarcinoma (LUAD) cohort have been widely reported. The integrated omics technologies revealed the molecular structure of LUAD and markers of tumor progression, which further elucidated new disease subtypes, signaling pathways, and provided potential targets for precision medicine in lung cancer [8–10].

LUAD is the main pathological type of XWLC. In recent years, the molecular mechanisms underlying the Xuanwei LUAD have been widely reported by high throughput techniques [11–13]. These studies described differentially expressed molecules and pathway changes in tumor tissues and provided high potential research value for XWLC. However, the exact mechanism of regulation and maintenance of protein expression and post-translational modifications (especially phosphorylation) and its relationship with XWLC is still unclear. In this study, we performed a comprehensive multi-omics approach, including transcriptome, proteome, and phosphoproteome, on fresh LUAD tissues and paired adjacent non-tumor tissues from three XWLC patients. We report the dysregulated RNA and protein molecular events in Xuanwei LUAD and the post-transcriptional regulatory mechanisms extensively involved by non-coding RNAs, as well as the signal transduction networks mediated by protein phosphorylation modifications. Besides, we combined our result with previously LUAD multi-omics datasets, which adds not only novel insights into the fundamental biological processes related to cancer but also provide interesting clues about potential therapeutic approaches for XWLC.

## METHODS

### Clinical specimens

Samples were collected from the Department of Thoracic Surgery I of Yunnan Cancer Hospital (the Third Affiliated Hospital of Kunming Medical University). This study was approved by the Ethical Committees of Yunnan Cancer Hospital (No. KY2019.57). We investigated three LUAD patients in the Xuanwei area from January 2019 to January 2020. Patients had undergone surgical resection and had not received any prior treatment, including chemotherapy or radiation. All patients used coal for heating or cooking for more than 10 years. The TNM stage was reviewed according to the 8th edition of the International Association for the Study of Lung Cancer (IASLC) staging system. Clinical information of individual patients, including age, histology, stage, and TNM, were listed in Supplementary Table 1. All patients provided informed consent.

Nearby tissue was designated as non-tumor and was greater than 5 cm away from the surgical margin. In total, six tissue samples (including three tumor tissues and adjacent non-tumor tissues) were taken from three patients. Each sample was cut into two pieces. One was stored in methanal for hematoxylin and eosin (HE) stain and transmission electron microscopy (TEM) examination. Another was stored in liquid nitrogen immediately and then used for omics experiments.

### Transcriptome sequencing

Total RNA from six tissue samples was extracted using a mirVana miRNA Isolation Kit (Ambion, Austin, TX, USA) following the manufacturer’s protocol. The quality and concentration of RNA were determined using an Agilent 2100 Bioanalyzer (Agilent Technologies, Santa Clara, CA, USA). After digesting the ribosomal RNA, samples with RNA Integrity Number (RIN) ≥7 were used to construct libraries by a TruSeq Stranded Total RNA with Ribo-Zero Gold. Paired-end sequences with 150 bp were generated on an Illumina HiSeq4000 platform. After removing adapters, clean reads were aligned to the human reference genome (hg38) to quantify coding genes, lncRNAs, and circRNAs. For small RNA, libraries were generated using NEBNext^®^ Multiplex Small RNA Library Prep Set for Illumina^®^ (NEB, Ipswich, MA, USA) and sequenced on an Illumina Hiseq2500 platform to get 50 bp single-end reads. The complete bioinformatics process can be seen in Supplementary Materials.

### Proteomic and phosphoproteomic experiment

Six tissue samples were transferred into a low protein binding tube and lysed with PMSF buffer to extract protein. Protein concentration was determined by BCA assay. After tryptic digestion and TMT labeling, the TMT-labeled peptide mix was fractionated using an Agilent Zorbax Extend C18 column on Agilent 1100 HPLC. Protein samples were separated on the Acclaim PepMap RSLC analytical column (RP-C18, Thermo Fisher Scientific, USA). Shotgun proteomics analyses were performed using Orbitrap Q Exactive HF-X mass spectrometer (Thermo Fisher Scientific). Protein with at least one unique peptide was identified at a false discovery rate (FDR) less than 1.0% on peptide or protein level. For phosphoproteomic samples, phosphopeptides were enriched using titanium dioxide beads (TiO2), and LC-MS/MS analysis was similar to proteomic experiment. The complete experimental and bioinformatics process can be seen in the supporting materials.

### Consistency analysis of omics data

Principal component analysis (PCA) and unsupervised cluster analysis were performed to evaluate the data accuracy and visualize differences among samples. Procrustes analysis was used to compare the overall relevance between omics data. Pearson correlation coefficients among RNA expression, protein expression, or phosphorylation intensity were calculated to assess the correlation of gene transcription, translation, and phosphorylation modification. All statistical analysis (including the following) was performed in the R program (v3.6.1) if there was no special software mentioned.

### Differential expression analysis and functional enrichment

DESeq2 (v1.26.0) [14] was applied for the detection of differentially expressed genes (DEGs) and other non-coding RNAs between tumor and non-tumor samples with a *P*-value of ≤0.05 and fold change (FC) of ≥2. The student’s *t*-test was performed with a permutation-based *P*-value of ≤0.05 and FC ≥1.2 to identify differentially expressed proteins (DEPs) or phosphosites in a pairwise manner. Gene Set Enrichment Analysis (GSEA) [15] was applied to find enriched pathways in transcriptome and proteome. Curated gene sets (H2) and GO gene sets (C5) were used for enrichment analysis, and an FDR value of 0.05 was adjusted as a cutoff.

### Molecular network construction

For understanding the physical association or co-expression patterns across DEPs, a protein-protein interaction (PPI) network of DEPs was established using the STRING database (v11.0) [16] with the highest confidence cutoff of 0.95. To explore the post-transcriptional regulation of non-coding RNAs, a competing endogenous RNA (ceRNA) network was constructed. TargetScan (v7.2) [17] was used to predict the coding genes targeted by miRNAs. LncRNAs or circRNAs-miRNA interactions were predicted using miRanda tools (v3.3) [18]. Network visualization was performed using Cytoscape software (v3.8.0) [19]. The Gene Ontology (GO) and Kyoto Encyclopedia of Genes and Genomes (KEGG) pathway enrichment were conducted to explore the function of network modules and target genes by using DAVID (v6.8) [20] and KOBAS (v3.0) [21] software, respectively. FDR ≤0.05 was recognized as significant enrichment.

### Kinase-substrate relationship prediction

The motif sequence represented the preference of kinase for substrates. We used MOMO (v5.1.1) [22] to analyze significantly enriched motifs. Kinase-substrate pairs were predicted by PhosphoSitePlus database (v6.5.9.3) [23] and NetworKIN algorithm (v3.0) [24]. Through the human kinome tree [25], we obtained the protein kinase domain sequences and constructed the phylogenetic tree.

### Public data analysis

Pan-cancer and tissue-specific analysis of significant prognostic factors were used in the transcriptome dataset from The Cancer Genome Atlas (TCGA) and Genotype-Tissue Expression (GTEx), respectively. Statistically significant differences were evaluated with the Student’s *t*-test. Kaplan-Meier survival analysis was performed to estimate the overall survival (OS) of LUAD patients and transcriptomic subtypes according to the TCGA dataset and the previous study [10]. Survival curves were calculated by Survival R package with the log-rank test *P*-value < 0.05. The main parameters used in the bioinformatical analysis were shown in Supplementary File 1.

### Data availability

RNAseq (mRNA, lncRNA, circRNA, and miRNA) data are available at GEO under accession number GSE165298. Other datasets used in the current study are available from the corresponding author on reasonable request.

## RESULTS

### Molecular profiling of Xuanwei LUAD

**Figure 1:**
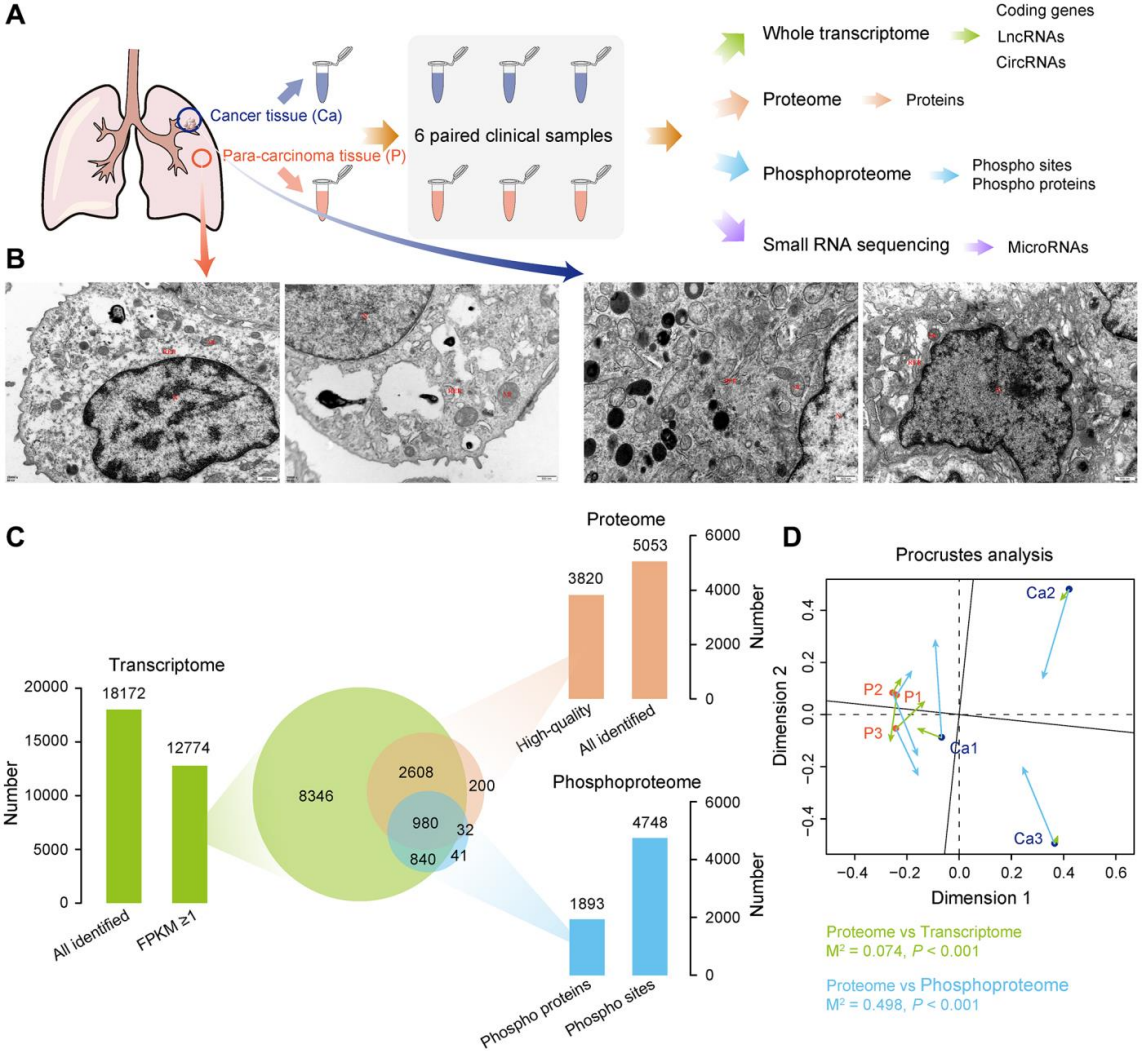
**Multi-omics landscape of LUAD samples.** (**A**) Experimental workflow to analyze transcriptome, proteome, phosphoproteome, and small RNA sequencing data. (**B**) Electron micrograph of the tissue section. Left, non-tumor tissue; right, LUAD tissue. (**C**) Quantitative statistics of total genes among transcriptome, proteome, and phosphoproteome, as well as the overlap of gene number among three omics. (**D**) Procrustes analysis shows the correlation between different omics.

In our study, we processed an integrative analysis of Xuanwei LUAD at the transcriptomics, proteomics, and phosphoproteomic level (Figure 1A). We first examined the characteristics of pathological tissues by TEM (Figure 1B). Compared with non-tumor tissues, the cell density in LUAD was higher, the nucleus was irregular polygonal, and the nucleoplasmic ratio was imbalanced. Most of the mitochondria in the cytoplasm were swelled, cristae were broken, dissolved, or even disappeared. A small amount of rough endoplasmic reticulum expanded and became cystic, and a small amount of autophagy and secondary lysosomes were also seen. Some cancer cells undergo apoptosis, with loss of cell volume, shrunk nucleus, and increased cytoplasmic electron cloud density.

A total of 82.33G clean reads were generated from whole-transcriptome sequencing with 90.26–90.98% Q30 bases distribution, and the average GC content were 49.05% (Supplementary Table 2). After clean reads were aligned with the human reference genome (hg38), the alignment efficiency was between 97.16% and 97.32%, suggesting good sequencing quality for all samples. Transcriptome analysis identified 12,774 genes with FPKMs of more than 1 (Supplementary Table 2). Meanwhile, proteomics measurement of six samples resulted in a total of 5,053 proteins based on Score Sequest HT 0 and unique peptide ≥1 (Supplementary Table 3A). After filtering, 3,820 high-quality proteins were identified (Supplementary Table 3B). Furthermore, a total of 4,748 phosphosites with high confidence corresponding to 1,893 phosphoproteins were recognized at a localization probability of ≥0.75 and delta score ≥8 (Supplementary Table 4). More than 90% of the high-quality proteins and phosphoproteins were overlapped with transcriptome, whereas a half number of phosphoproteins were also identified in the proteome (Figure 1C). This high-quality data was used in further analyses.

The PCA of transcriptomic, proteomic, and phosphoproteomic data revealed a significant difference between LUAD and non-tumor samples (Supplementary Figure 1A–1C), and Procrustes analysis revealed a considerable correlation among three omics (Figure 1D). These results provide an opportunity to explore the consistent association among three omics data.

### Integrative transcriptome and proteome analysis

**Figure 2:**
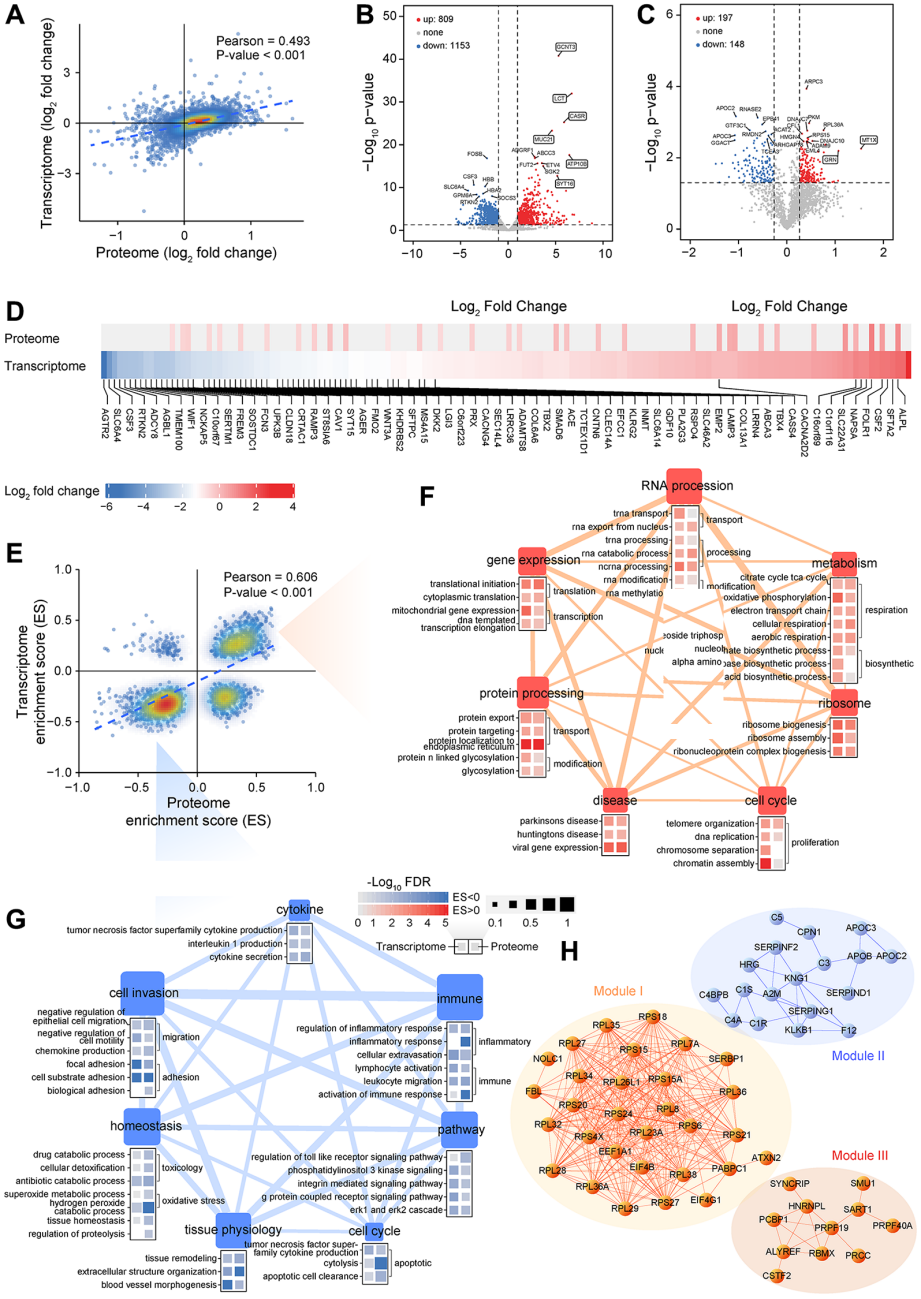
**Integrative analysis of transcriptomics and proteomics data generated from LUAD samples.** (**A**) Correlation between RNA expression and protein expression. (**B**, **C**) Analysis of differentially expressed genes between LUAD and non-tumor samples at transcriptional (**B**) or translational level (**C**). (**D**) Fold change in RNA and protein levels of lung signature-associated genes. (**E**) Correlation between transcriptome and proteome gene set enrichment scores. (**F**, **G**) Analysis of biological processes or pathways that are significantly activated (**F**) or inhibited (**G**) in LUAD based on GSEA. (**H**) Interaction network analysis of DEPs highlights three functional modules.

An integrated analysis of transcriptome and proteome changes was performed to investigate the extent and levels of transcriptional or translational regulation. There were 3,588 genes shared between transcriptome and proteome (Figure 1C). By comparing the expression level of mRNAs and proteins, we observed most of these shared genes had a strong positive correlation (Figure 2A), which means that two omics data changed synchronously. Functional analysis also showed that almost all significantly enriched terms were in a similar state of activation or inhibition in two datasets (Supplementary Figure 2A, 2B). Activated pathways involved in metabolism, genetic information processing, and cellular processes, which accelerate the proliferation of tumor cells. Cell adhesion is an essential process for tumor metastasis, and decreased cell adhesion is associated with cancer spread [26]. Extracellular matrix-related terms were also downregulated. It was reported previously that breaking the extracellular matrix around the tumor is necessary for tumor cell migration and invasion [27]. Interestingly, the gene set involved in the immune system had a large mRNA score but only a small protein score, indicating that these proteins may tightly be controlled at a post-transcriptional level to have suitable amounts for tumor progression.

A total of 1,962 genes were differentially expressed between LAUD and non-tumor samples, 809 of which were upregulated and 1,153 were downregulated (Figure 2B). Several abnormally highly expressed genes were observed, and some of them have been proven to have essential roles in various cancers. For instance, GCNT3 is a novel core mucin synthase, usually highly expressed in non-small-cell lung cancer (NSCLC) and correlated with tumor invasion [28]. CASR controls body calcium homeostasis, and its overexpression can induce osteoclast differentiation and promote bone metastasis in LUAD [29]. Proteomic analysis revealed 197 upregulated and 148 downregulated DEPs in LUAD (Figure 2C). A group of significantly upregulated proteins was also known to be related to lung cancer progression. It is well known that MT1X is one of the isoforms of metallothionein. Theocharis et al. observed that metallothionein expression was prominent in squamous cell lung carcinoma and adenocarcinoma [30]. GRN implicates in tumorigenesis as an autocrine growth and survival factor [31], and is also reported being a prognostic factor in localized NSCLC [32]. Moreover, a lot of previously reported lung signature genes [33] also had significant differential expression in our LUAD samples (Figure 2D). The above results generate a vast resource of dysregulated molecular events, which provide valuable clues for recapitulating the potential molecular regulation or metabolic network of LUAD.

However, only a few differentially expressed genes were shared between transcriptome and proteome (Supplementary Figure 2C). To compare similarities or differences of functional changes at the transcription and translation level, an unbiased analysis of gene expression changes was performed using GSEA. Likewise, enrichment scores were in a high degree of significant consistency (Figure 2E). Several categories involved in metabolism, molecular processes, homeostasis, cell behavior, and disease-related pathways were associated with cancer hallmarks (Figure 2F, 2G). Many functions related to intracellular homeostasis and tissue physiology were disordered and contributed to a cascade of dysfunctional processes such as inflammation, stress response, and immune-suppressive.

PPI network analysis identified three necessary modules (Figure 2H and Supplementary Figure 2D–2G). Interestingly, all proteins in module I and III were upregulated in LUAD, whereas module II was composed of downregulated proteins, suggesting the biological co-activation or co-inhibition are general. Functional enrichment analysis revealed that module I and III are associated with ribosome, gene expression, and RNA process, including splicing, transport, metabolism, and degradation. In contrast, module II is mainly involved in the protein process and response to stimuli (Figure 2H). Strikingly, nodes at the core of the network involved various ribosomal proteins (RPs) that were highly expressed in LUAD (Figure 2H). RPs exert a vital role in ribosome biogenesis and cell cycle, and increased ribosomal activity is an essential feature of tumorigenesis [34]. In the down-regulated protein module, core protein KNG1 is known associated with tumorigenesis and has been reported as a novel biological fluid biomarker in lung squamous cell carcinoma [35]. A2M inhibits tumor cell adhesion and migration by impeding β-catenin signaling [36], also downregulated in NSCLC and as a core node in the PPI network [37].

### Characterization of non-coding RNAs and their regulatory roles

**Figure 3:**
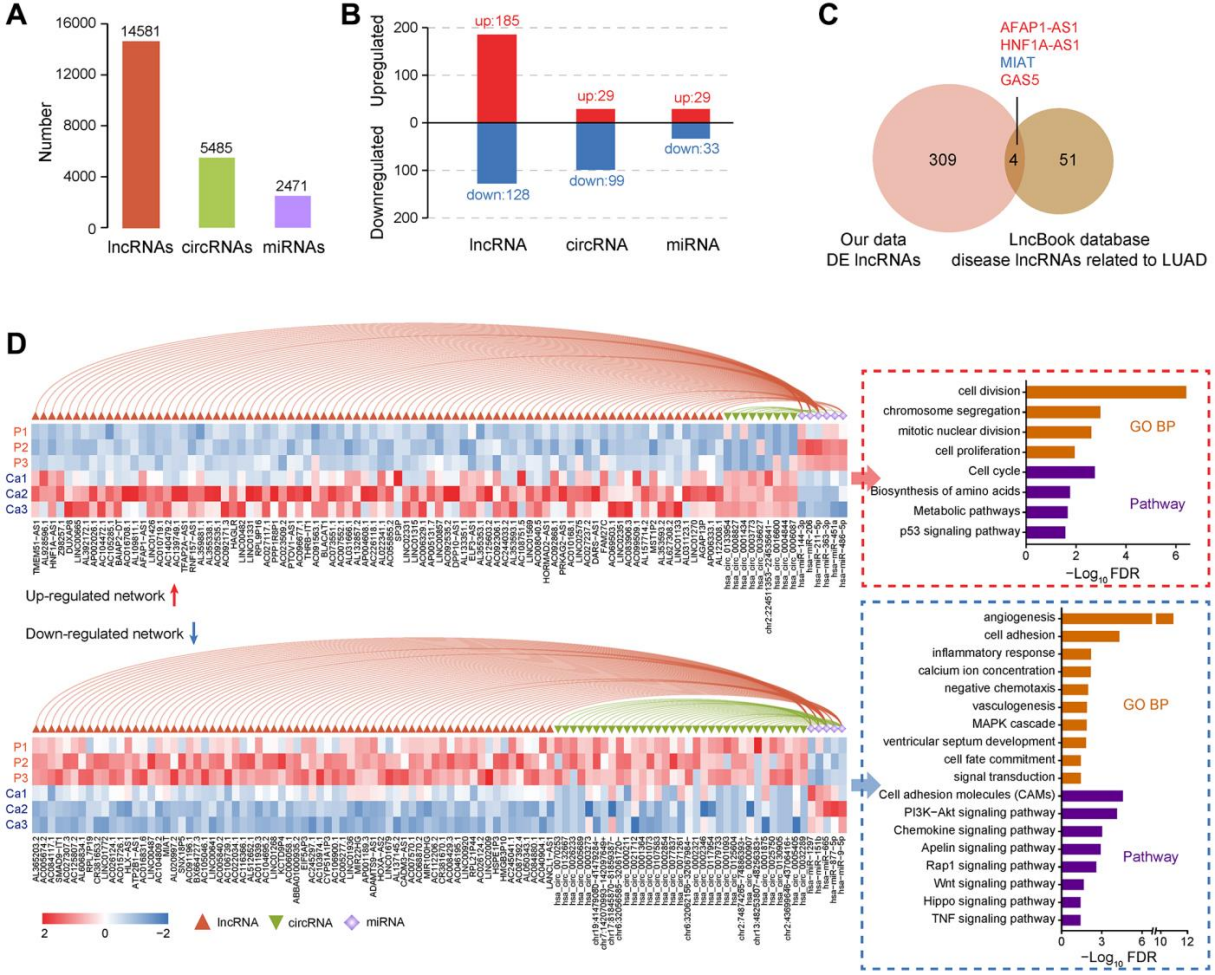
**Identification and functional analysis of non-coding RNAs in LUAD.** (**A**) Quantitative statistics of lncRNAs, circRNAs, and miRNAs. (**B**) Analysis of differentially expressed lncRNAs, circRNAs, and miRNAs between LUAD and non-tumor samples. (**C**) Annotation of LUAD-related lncRNA markers based on the LncBook database. (**D**) The relationship between miRNAs and lncRNAs or circRNAs, expression of non-coding RNAs in each sample, and the functional enrichment of downstream target genes.

Extensive inconsistencies were also present between transcriptome and proteome (Figure 2A, 2E). One main reason is that the detection capability of different omics has its limitation. The various forms of gene expression products cannot be guaranteed to be covered at the same time. Post-transcriptional regulatory event is another critical situation, such as non-coding RNAs, which can cause significant differences in transcription and protein level. According to our transcriptome data, a total of 14,581 lncRNAs, 5,485 circRNAs, and 2,471 miRNAs were identified among six samples, respectively (Figure 3A). We then analyzed differentially expressed non-coding RNAs between two groups, and the results were shown in Figure 3B.

Numerous studies have demonstrated that non-coding RNAs play vital roles in varieties of biological processes and are closely related to the occurrence of multiple human diseases. Based on the LncBook database [38], we identified four lncRNAs that were confirmed as LUAD markers (Figure 3C and Supplementary File 2). Upregulated AFAP1-AS1 is closely related to the metastasis and poor prognosis of lung cancer cells. AFAP1-AS1 regulates lung cancer proliferation, migration, and invasion through the competitive upregulation of RRM2 to inhibit miR-139-5p, IRF7, and RIG-I-like receptor signaling pathways, or epigenetic inhibition of p21 expression [39–41]. Similarly, the regulation of HNF1A-AS1 can be mediated by miRNAs, such as contacting miR-17-5p to promote the proliferation and invasion of lung cancer cells [42, 43]. MIAT can target the miR-149-5p/FOXM1 axis to regulate lung cancer progression partially [44]. GAS5 affects the occurrence and invasion of lung tumors through sponge miR-23a or miR-135b [45, 46].

It seems that miRNAs extensively mediate the regulation of target genes. We further constructed ceRNA regulatory networks and identified a group of key regulatory axes (Figure 3D and Supplementary Figure 3). Most of these crucial miRNAs have been reported to implicate in the pathological process of cancer biology. MiR-144-3p and miR-206 can inhibit TGF-β signaling, thereby restricting tumor growth and metastasis of LUAD [47, 48]. MiR-363-3p suppresses lung tumor cells migration and invasion through epithelial-mesenchymal transition inhibition [49]. In the upregulated ceRNA network, overexpressed lncRNAs or circRNAs bind these miRNAs to disable their functions, activating downstream cell proliferation, cycle, metabolism, and p53 pathways (Figure 3D and Supplementary File 3). Likewise, for the downregulated ceRNA network, unsuppressed miRNAs may also be high-risk factors. Such as miR-1297 promotes the proliferation of non-small cell lung cancer cells by participating in the PTEN/Akt/Skp2 signaling pathway [50]. Downregulated downstream target genes in the network mainly involved cell adhesion, inflammation, chemotaxis, and apoptosis signals, facilitating tumor cell migration and invasion (Figure 3D). Interestingly, since the ceRNA mechanism mainly represented post-transcriptional regulation, this phenomenon may also partially explain the differences in immune system-related pathway alters observed between transcriptome and proteome (Supplementary Figure 2A). In summary, dysregulated non-coding RNAs can directly or indirectly regulate the function of coding genes to accelerate the pathological process of LUAD.

### Analysis of the phosphoproteomic features

Protein phosphorylation plays an essential role in the activation and inactivation of protein functions, including enzymes involved in metabolism, transcription factors that initiate gene expression, or protein complexes that determine cell fate. We then quantified and characterized the global profile of the phosphoproteome.

**Figure 4:**
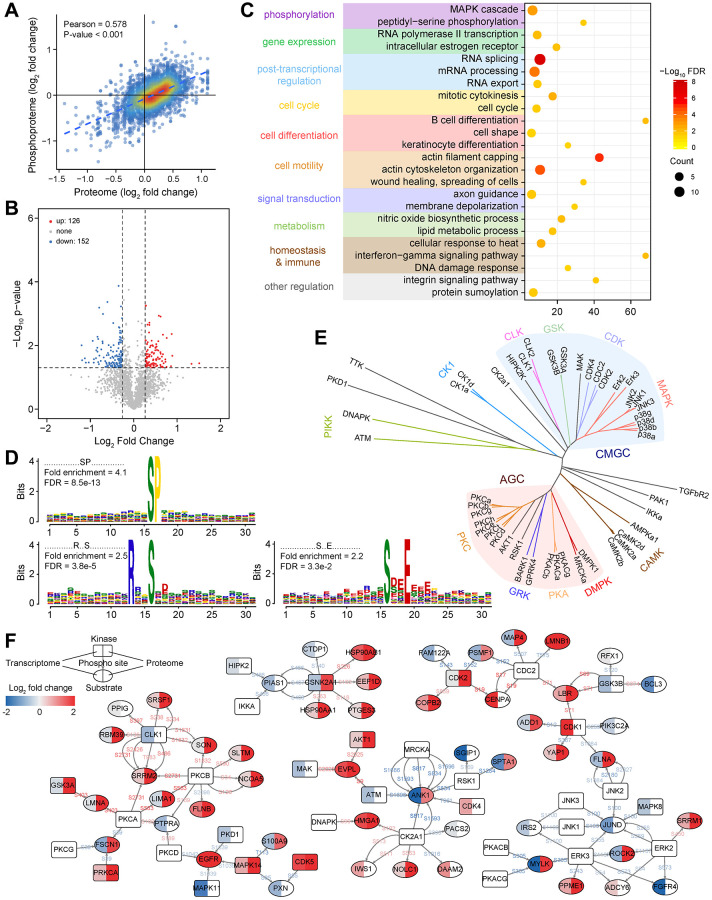
**Functional characterization of phosphorylated proteins and construction of the kinase-substrate regulatory network.** (**A**) Correlation between protein expression and its phosphorylation intensity. (**B**) Changes in the intensity of phosphosites between LUAD and non-tumor samples. (**C**) Functional enrichment analysis of phosphorylated proteins with differential phosphorylation. (**D**) Significantly enriched kinase motifs for phosphosites that are dysregulated in LUAD. (**E**) Kinase annotation and family analysis for dysregulated phosphosites. (**F**) A phosphorylation regulatory network constructed based on dysregulated phosphosites and annotated kinase-substrate relationship pairs in LUAD.

Only 4,748 high-quality phosphosites were considered for further analysis (localization probability ≥0.75 and delta score ≥8, Figure 1C). More than half of phosphoproteins had only a single phosphorylation site, about a quarter of phosphosites were doubly phosphorylated, as well as only a fraction was triply (or more) phosphorylated (Supplementary Figures 4A and 5A). The majority of phosphorylation events occurred on serine (91.03%), followed by threonine (8.36%), whereas less than 1% of phosphotyrosines were detected (Supplementary Figures 4B and 5B). By comparing with the modification sites from the PhosphoSitePlus database, 86.57% of our quantified phosphosites were identical (Supplementary Figures 4C and 5C). However, only 8.22% were annotated as regulatory sites (Supplementary Figures 4D and 5D), implying that the functional role of most phosphorylation events quantified here had not been investigated. Interestingly, phosphorylation sites with known regulatory functions were generally involved in processes or signaling pathways related to transcription, cell proliferation, migration, apoptosis, and carcinogenesis (Supplementary Figures 4E–4I and 5E). This suggested that abnormal activation or inhibition of downstream target genes or signal axises caused by altered protein phosphorylation state may be necessary causes of LUAD. A higher correlation between phosphosites activation and protein expression was found (Figure 4A) for the 1,012 phosphoproteins detected simultaneously by proteome and phosphoproteome (Figure 1C). To further confirm whether protein phosphorylation was related to disease, we analyzed the phosphosite intensity difference between LUAD and non-tumor samples. In total, 126 phosphosites from 90 proteins were increased, and 152 phosphosites from 117 proteins were decreased (Figure 4B and Supplementary Figure 5F). Similarly, hierarchical clustering analysis resulted in two separate groups based on the increased and decreased phosphosites (Supplementary Figure 5G–5I). Functional enrichment analysis showed that these phosphoproteins played significant roles in gene expression regulation, RNA and protein modification, cell cycle and motility, metabolism, and physiological regulation related processes or pathways (Figure 4C).

By analyzing the amino acid enrichment around differentially accumulating phosphosites, we found three significantly enriched kinase motifs belonging to the phosphoserines motif (Figure 4D). The enrichment of phosphothreonine or phosphotyrosine motifs were not observed, which may be because of their low abundance. Through PhosphoSitePlus and NetworKIN to predict the kinase-substrate relationship pairs, members of specific kinase families were highlighted in LUAD (Figure 4E). In particular, the activities of the AGC and CMGC group were more noticeable, mainly including nine members of the MAPK family, seven members of the PKC family, three members each from the PKA and CDK family.

To better understand the phosphorylation in LUAD, we focused on significantly different phosphate sites and constructed a kinase-substrate relationship network (Figure 4F). The unique network contained 85 proteins corresponding to 109 relationships and showed their changes in the RNA expression, protein expression, and phosphorylation response. We noticed that in most cases, kinase or substrate activities were highly consistent with the level of phosphorylation. Specific kinases were involved in regulating multiple biological processes, and many substrates were also involved in crosstalk between kinases, indicating that there was a complex network in the body rather than a linear cascade of signal transduction pathways. For instance, SRRM2 had 6 significant upregulated phosphosites with higher activation in LUAD. SRRM2 has been identified as a scaffold protein for splicing factors [51], and its overexpression may cause splicing-based damage to cell cycle-related genes and cause cell abnormalities. JUND is an influential transcription factor regulating cell apoptosis and resist oxidative stress by regulating genes involved in antioxidant defense and hydrogen peroxide production [52]. Our data showed that the phosphorylation level of JUND was generally inhibited, which would lead to uncontrolled cell proliferation and defects in the ability to respond to stress. These results all revealed that kinase signal dysregulation contributed to the progress of LUAD.

### Comparative omics analysis of multiple LUAD datasets

We also compared our dataset with previous transcriptome, proteome, or phosphoproteome studies involved in LUAD research, including TCGA database, Clinical Proteomic Tumor Analysis Consortium (CPTAC) database, the large-scale LUAD dataset for the Chinese population (GSE140343, transcriptome; IPX0001804000, proteome, and phosphoproteome) [10], and another Xuanwei LUAD dataset (GSE89039, the only transcriptome was available) [53].

**Figure 5:**
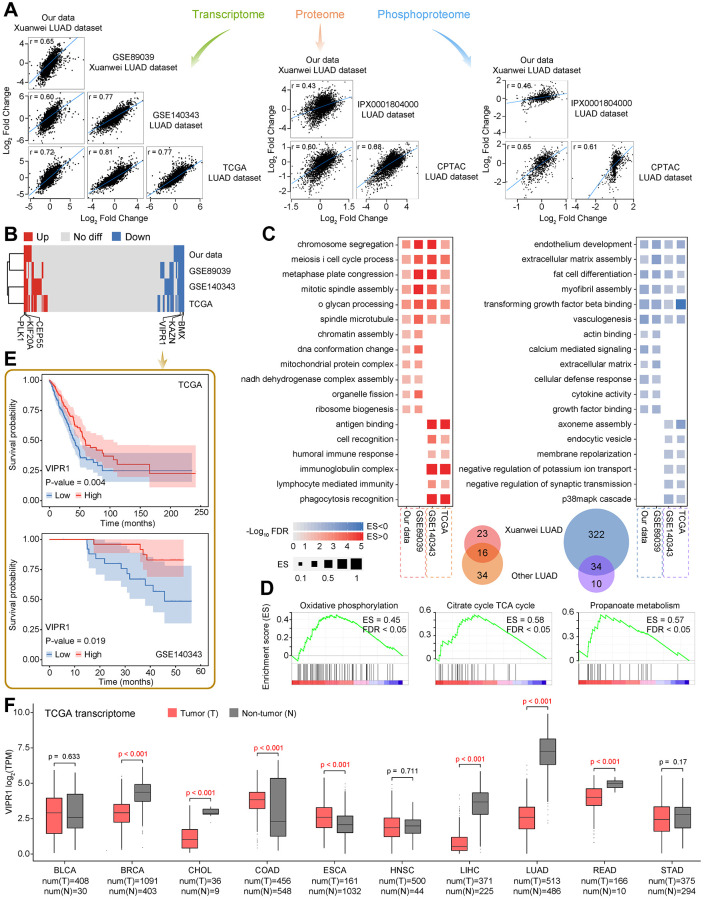
**Comparative analysis of public data of LUAD expression profile.** (**A**) The correlation between our dataset and previously published transcriptome, proteome, and phosphoproteome datasets of LUAD. (**B**) The heat map shows the dysregulated state of common genes among four transcriptome datasets. The label highlights six significant prognostic factors. (**C**) GSEA of four transcriptome datasets, the representative biological processes that are significantly activated or inhibited are shown. (**D**) Three representative metabolic pathways that are significantly activated in Xuanwei LUAD proteome by using GSEA. (**E**) Kaplan-Meier curves show the relationship between the overall survival time of LUAD patients and VIPR1 expression level in tumors according to publicly available LUAD datasets. (**F**). The expression of VIPR1 among pan-cancer settings.

A high homogeneity was observed across all datasets, suggesting that the overall changes in RNA or protein among different sources of LUAD were similar (Supplementary Table 5, Figure 5A). The hierarchical clustering of the dysregulated status of shared genes among four datasets revealed that the two Xuanwei LUAD datasets had closer similarities, highlighting that Xuanwei LUAD was more specific than other regions in China and abroad (Figure 5B). Hardly any gene was observed to have an opposite up-down regulation state in the comparison. GSEA revealed that biological processes related to the cell cycle were simultaneously activated among four LUAD groups. In contrast, functional activities of cell differentiation and tissue development were inhibited, which were general features of tumor cells (Figure 5C). In contrast, chromosomal components and energy production seem to have higher activity in Xuanwei LUAD. The GSEA of the proteome revealed that a similar series of crucial metabolic pathways were highly activated in Xuanwei LUAD (Figure 5D–5F), but not significant in CPTAC and Chinese large-scale LUAD datasets. These results are implying that Xuanwei LUAD may experience more severe energy disorders. Notably, we observed pervasive downregulated functional items were appeared in Xuanwei LUAD, including cell recognition, immunity, cytokines, as well as signal transduction (Figure 5C). Combining these characteristics, we speculated that the accompanying more molecular deficiencies might be a vital factor of the high LUAD incidence in Xuanwei.

We further investigated possible biomarkers in Xuanwei LUAD. Combining the transcriptome and overall survival of patients from TCGA and Chinese large-scale LUAD datasets, a total of 6 significant prognostic factors were identified in 127 upregulated, and 275 downregulated common genes from all four transcriptome datasets (Figure 5B and Supplementary Figures 6 and 7). Two large-scale LUAD datasets consistently showed that patients with higher expression of CEP55, KIF20A, or PLK1 had a worse prognosis, whereas lower expression of BMX, KAZN, or VIPR1 would bring higher risks. Interestingly, these six prognostic factors were also continuously activated or inhibited in almost all types of tumors according to the pan-cancer analysis of TCGA transcriptome data (Figure 5F, Supplementary Figures 8 and 9), implying that they can be used as general tumor biomarkers.

### The expression characteristics of VIPR1 in LUAD patients

**Figure 6:**
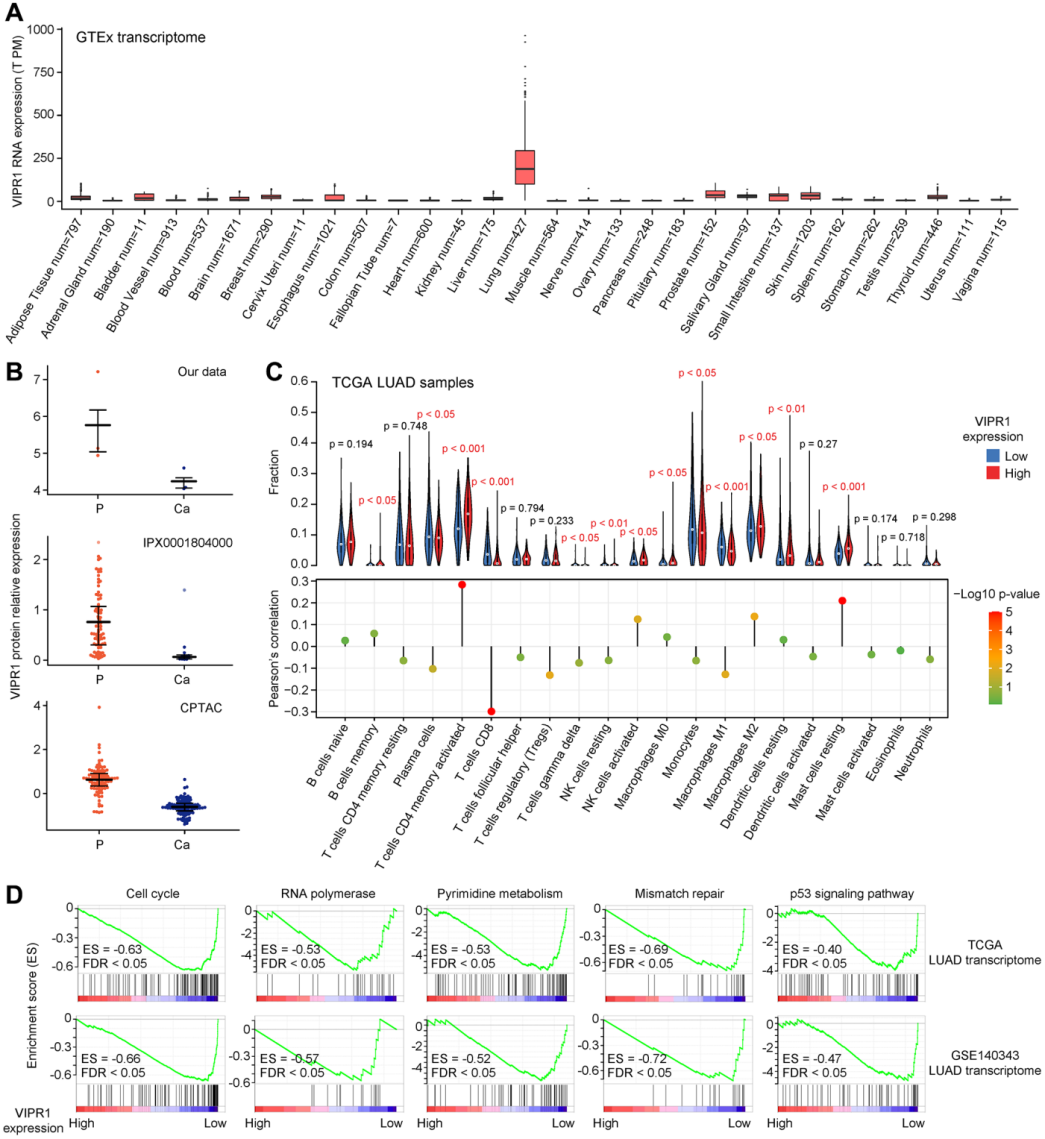
**Analysis of VIPR1 expression characteristics in LUAD.** (**A**) The tissue-specific expression of VIPR1 mRNA from GTEx. (**B**) Relative protein expression levels of VIPR1 in three LUAD proteomic data sets. (**C**) GSEA of the transcriptome of patients with high VIPR1 expression relative to patients with low VIPR1 expression according to TCGA and Chinese large-scale LUAD datasets. (**D**) Five representative pathways that are significantly enriched in the low VIPR1 expression group are shown.

It was worth noting that VIPR1 showed a strikingly high level in lung tissue when we checked the tissue-specific expression of these six prognostic-related genes through the GTEx database (Figure 6A). Consistent with the transcriptome, the proteome datasets also showed that the protein expression of VIPR1 was reduced in LUAD (Figure 6B). These highlighted the important function of VIPR1 in the lung. Related studies have confirmed that VIPR1 has a significant inhibitory effect on the growth and development of LUAD cells. Knockdown of VIPR1 increases the growth, migration, and invasion of LUAD cells [54–57]. GSEA of TCGA LUAD transcriptome data showed that pathways related to cell cycle, gene expression, metabolism, DNA damage, and cancer were more significantly enriched in low expressing VIPR1 patient group (Figure 6C, 6D). This was very similar to our Xuanwei LUAD molecular characteristics (Figure 2F) and gave patients with low VIPR1 expression a worse prognosis. Collectively, we provided more insights into VIPR1 as a LUAD general biomarker.

## DISCUSSION

Xuanwei is one of the regions with the highest incidence and mortality of lung cancer in China. The incidence of XWLC in women is as high as 400/100,000, which is 20 times higher than the national average incidence of lung cancer. However, its etiology and pathogenesis are complicated and are still mostly unknown, especially the lack of knowledge about protein expression and modification. The occurrence and development of XWLC is a very complex multi-gene event, involving the functional changes of multiple oncogenes and tumor suppressor genes. However, studies on the expression profile at whole proteome and transcriptome-wide in the carcinogenesis and progression of XWLC adenocarcinoma are rarely reported. In this study, we reported the vast landscape of transcriptome, proteome, and phosphoproteome of Xuanwei LUAD for the first time. We systematically characterized the differential molecular processes in lung tissue to provide more insights into regional lung cancer.

The significant correlation between transcriptome and quantitative proteome proved the reliability of high-throughput and mass spectrometry detection capabilities. Tang et al. found that protein-mRNA consistency in breast tumors was enhanced and could be used as a novel disease characteristic and prognostic factor [58]. Therefore, we believe that the positive correlation between transcriptome and proteome also highlights the progress of Xuanwei LUAD. We examined potential differences between transcriptome and proteome analysis by using non-cancerous tissue pairs adjacent to tumors and together looked at the connections between differentially expressed mRNA and proteins and their pathways. The dysregulated genes and proteins contained many known lung signatures, indicating a severe loss of lung tissue function. However, there were differences at the individual molecule level due to the sensitivity of different omics detection methods or extensive post-transcriptional or translational level adjustments. Therefore, omics joint analysis can provide tumor biology insights missed by a single omics. The integrated study consistently showed abnormal metabolism, immune loss, increased cell proliferation and invasion, and inhibition of apoptotic pathways in Xuanwei LUAD, all of these were general signs of cancer.

We identified a mass of unique non-coding RNAs, including lncRNAs, circRNAs, and miRNAs, further expanded the genome resources of Xuanwei LUAD. Partially dysregulated lncRNA had been described as well-known LUAD markers. The ceRNA network highlighted a set of core miRNAs and reveals the extensive post-transcriptional regulation mechanism of non-coding RNAs, emphasizing that non-coding RNAs coordinate the pathological process of LUAD by directly or indirectly regulating the function of coding genes. In addition, target genes also explained to some extent the observed molecular differences between transcriptome and proteome.

In this study, we quantified the phosphoproteins in Xuanwei LUAD for the first time and identified many novel phosphosites. Comprehensive phosphoproteome analysis provided supplementary information on pathway activity. Even components in the pathway have not changed in mRNA or protein expression, the phosphorylation status during signal transduction will also affect downstream signal activation. We provided evidence support for members of the core kinase family and constructed a kinase-substrate network to report necessary protein modifications and possible regulatory modes of phosphorylation cascades. The role of kinase pathways in cancer, immune, or homeostasis disorders diseases still requires further attention. For example, the MAPK are prevalent in non-small cell lung cancers, e.g., adenocarcinoma, while relatively suppressed in small cell lung cancer, which suggested that the subtype specific pathways in LUAD. Proper targeting of these signal axes may delay or cancel the progression of LUAD.

Finally, we compared our Xuanwei LUAD data with the previous large-scale LUAD datasets. There was a high correlation among multiple omics datasets, indicating the overall similar molecular patterns of LUAD. In particular, Xuanwei LUAD showed a higher degree of activation of the aerobic breathing pathway. Previous studies have pointed out that cellular metabolic reprogramming caused by activated mitochondrial oxidative phosphorylation and TCA cycle are signs of high tumor progression [59]. When it is beneficial to meet tumor energy requirements, the maintenance of OXPHOS transcription levels similar to those seen in normoxic cells can provide a mechanism to induce oxidative phosphorylation activity rapidly [60]. Genes encoding metabolic functions tend to show a high protein-mRNA correlation, emphasizing that cancer cells require stricter metabolic regulation to survive by linking transcription and translation [58]. Thus, Xuanwei LUAD patients may benefit from a therapy directed against metabolic modulation of intrinsic tumor pathways (redox). VIPR1 was particularly concerned because of its unique expression status in lung tissue. By supplementing the insights into the molecular characteristics of VIPR1-deficient patients, we also emphasized the possible therapeutic prospects of metabolic therapy in VIPR1-deficient patients, including Xuanwei LUAD.

Overall, our results revealed the molecular structure and tumor progression markers of Xuanwei LUAD and may provide precise medical approaches for treatment. This study provides a foundation for the follow-up in-depth analysis of the mechanism in XWLC.

## Supplementary Materials

Supplementary Figures

Supplementary Table 1

Supplementary Table 2

Supplementary Table 3

Supplementary Table 4

Supplementary Table 5

Supplementary File 1

Supplementary File 2

Supplementary File 3
