# Supplementary material for: Integrated multi-omics analysis for lung adenocarcinoma in Xuanwei, China
Source: Aging (Albany NY). 2023 Dec 13;15(23):14263–91. doi: 10.18632/aging.205300 (PMC10756121; doi:10.18632/aging.205300)
Supplement: Supplementary Figures [file aging-15-205300-s001.pdf]

SUPPLEMENTARY FIGURES

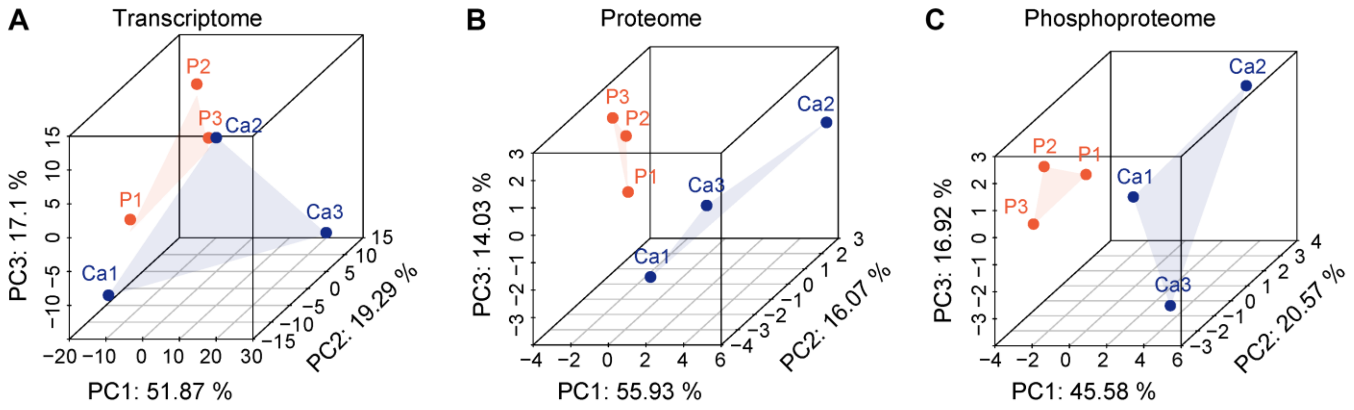

Supplementary Figure 1. Principal component analysis of transcriptome (**A**), proteome (**B**), and phosphoproteome data (**C**) shows the similarity and reproducibility among different biological replicates, related to Figure 1.

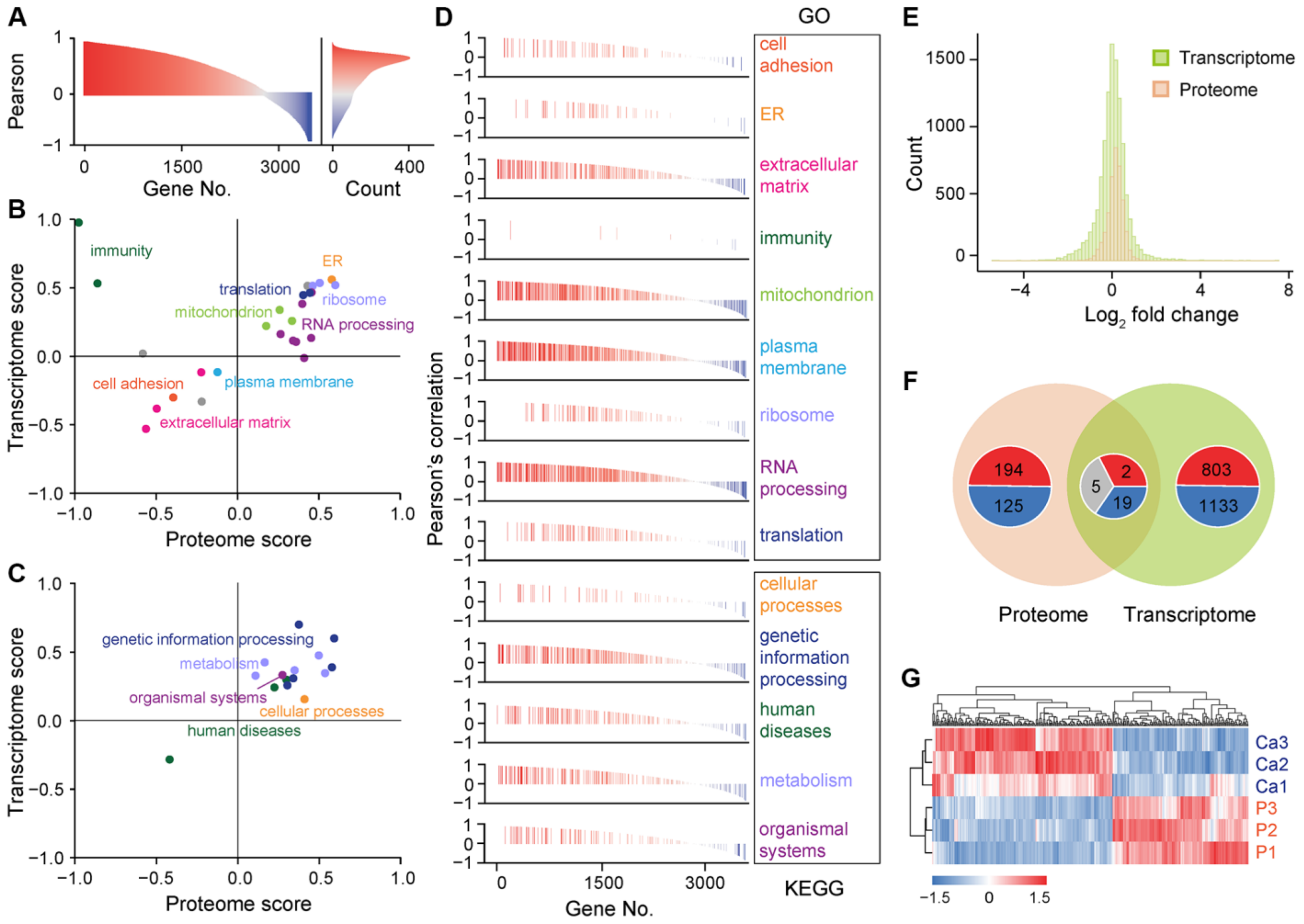

Supplementary Figure 2. Analysis of transcriptome and proteome consistency and differentially expressed genes and proteins, related to Figure 2. (**A**) The distribution of the Pearson correlation coefficient of RNA and protein expression values of shared genes between two omics data. (**B**, **C**) 2D annotation enrichment analysis examines the consistency of biological processes (**B**) or pathways (**C**) of shared genes between two omics data-based enrichment score. (**D**) The distribution of the Pearson correlation coefficient of RNA and protein expression values in each significantly enriched function. (**E**) The distribution of the fold change of RNAs and proteins. (**F**) Intersection of differentially expressed genes between transcriptome and proteome. For each pie chart, red represents upregulation genes, blue represents downregulation genes, and gray represents genes with opposite up-down trends between groups. (**G**) Heat map shows hierarchical clustering of differentially expressed proteins.

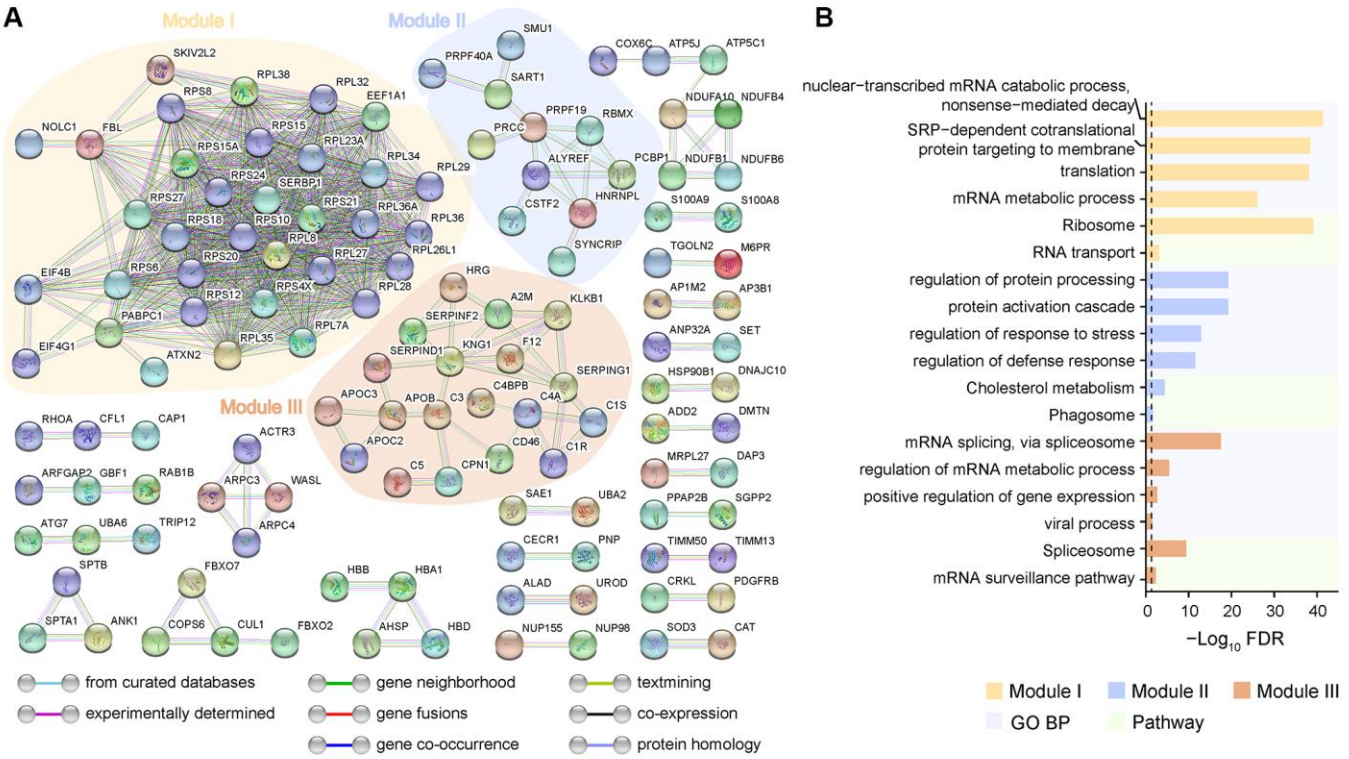

Supplementary Figure 3. Protein-protein interaction (PPI) network of differentially expressed proteins, related to Figure 2. (**A**) STRING network visualizing the functional protein association in LUAD at confidence probability of 0.95. (**B**) Functional enrichment analysis of proteins in key network modules I, II and III.

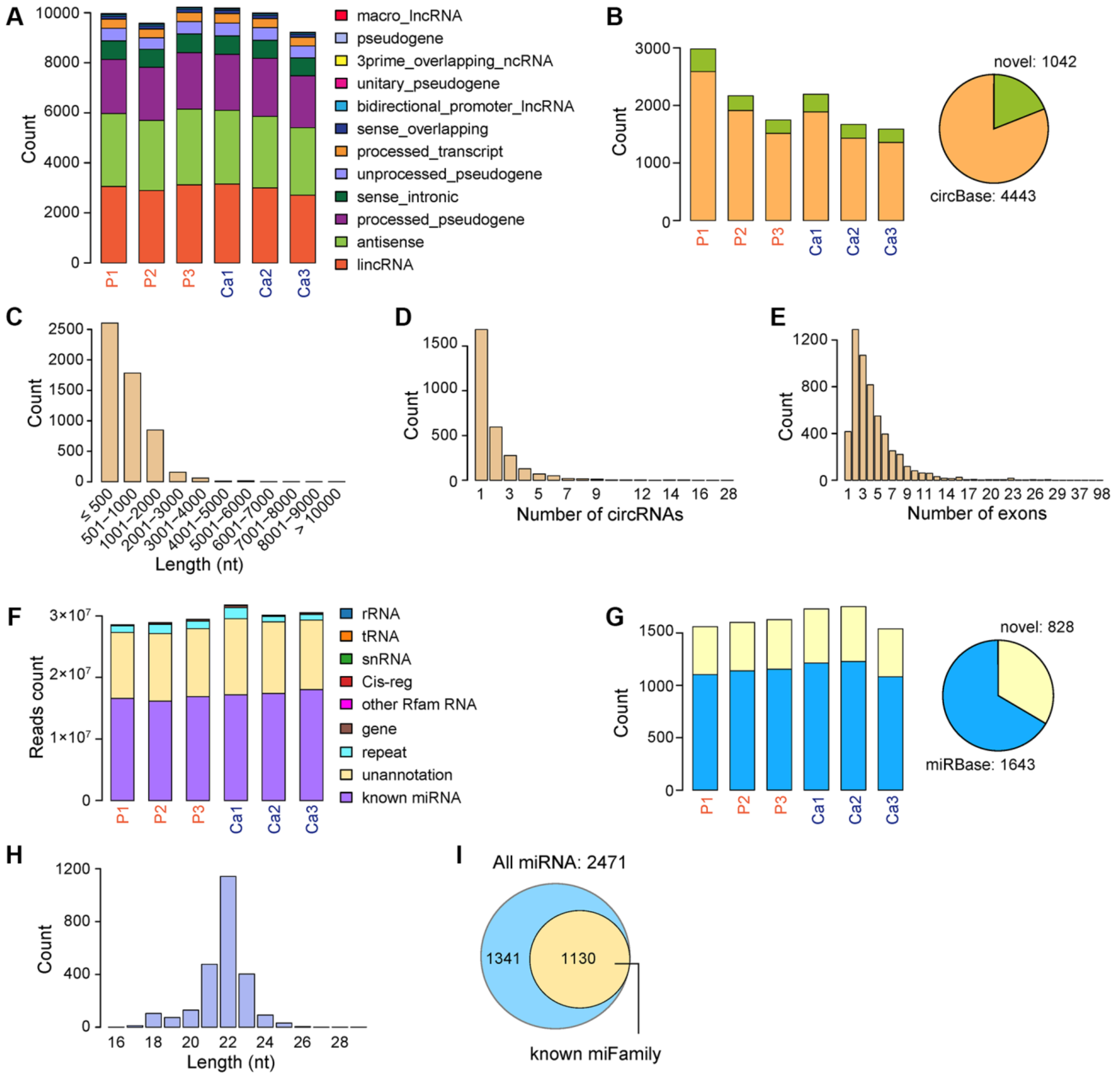

Supplementary Figure 4. Identification and characterization of non-coding RNAs in LUAD, related to Figure 3. (**A**) Types and quantity statistics of lncRNAs based on FPKM ≥1. (**B**) The number of unique circRNAs detected and its comparison with the circBase database. (**C**) Length distribution of circRNAs. (**D**) Number of circRNAs produced by unique gene. (**E**) Exon number distributions of circRNAs. (**F**) Number of reads from different types of small RNA sources in the genome. (**G**) The number of unique miRNAs detected and its comparison with the miRBase database. (**H**) Length distribution of miRNAs. (**I**) Family statistical analysis on the detected miRNAs.

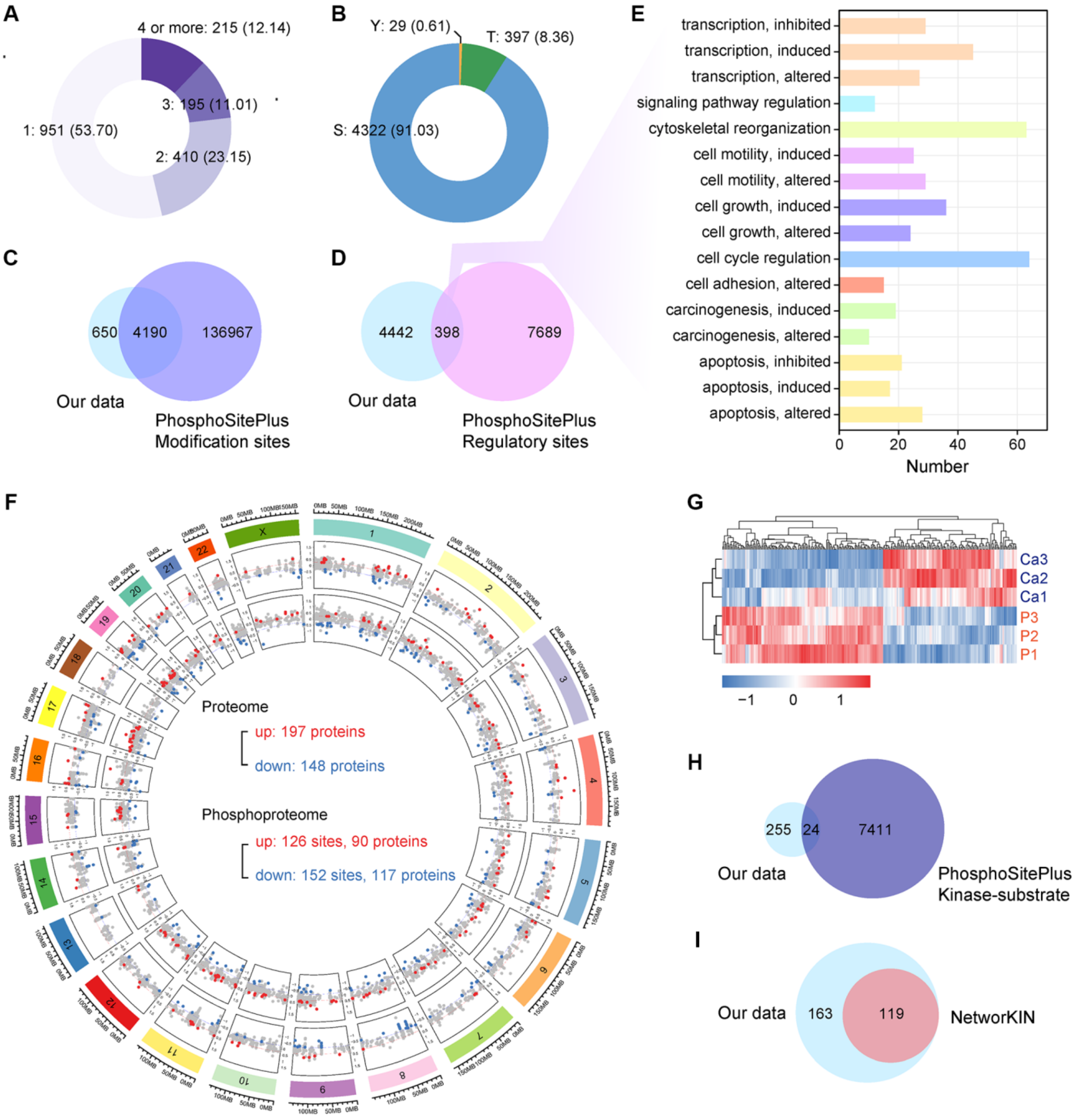

Supplementary Figure 5. Identification and characterization of phosphosites in LUAD, related to Figure 4. (**A**) Number of phosphosites from unique protein. (**B**) Types and quantity statistics of phosphorylation events. (**C**, **D**) Number of phosphosites and its comparison with the modification sites (**C**) or regulatory sites (**D**) from PhosphoSitePlus database. (**E**) Regulatory function annotations of known phosphosites. (**F**) Genomic distribution of proteins with differential expression and phosphorylation in LUAD. (**G**) Heat map shows hierarchical clustering of phosphosites with differential phosphorylation. (**H**, **I**) Number of phosphosites that are deregulated and its comparison with the kinase-substrate dataset from PhosphoSitePlus (**H**), or predict potential kinase-substrate relationship by using NetworKIN (**I**).

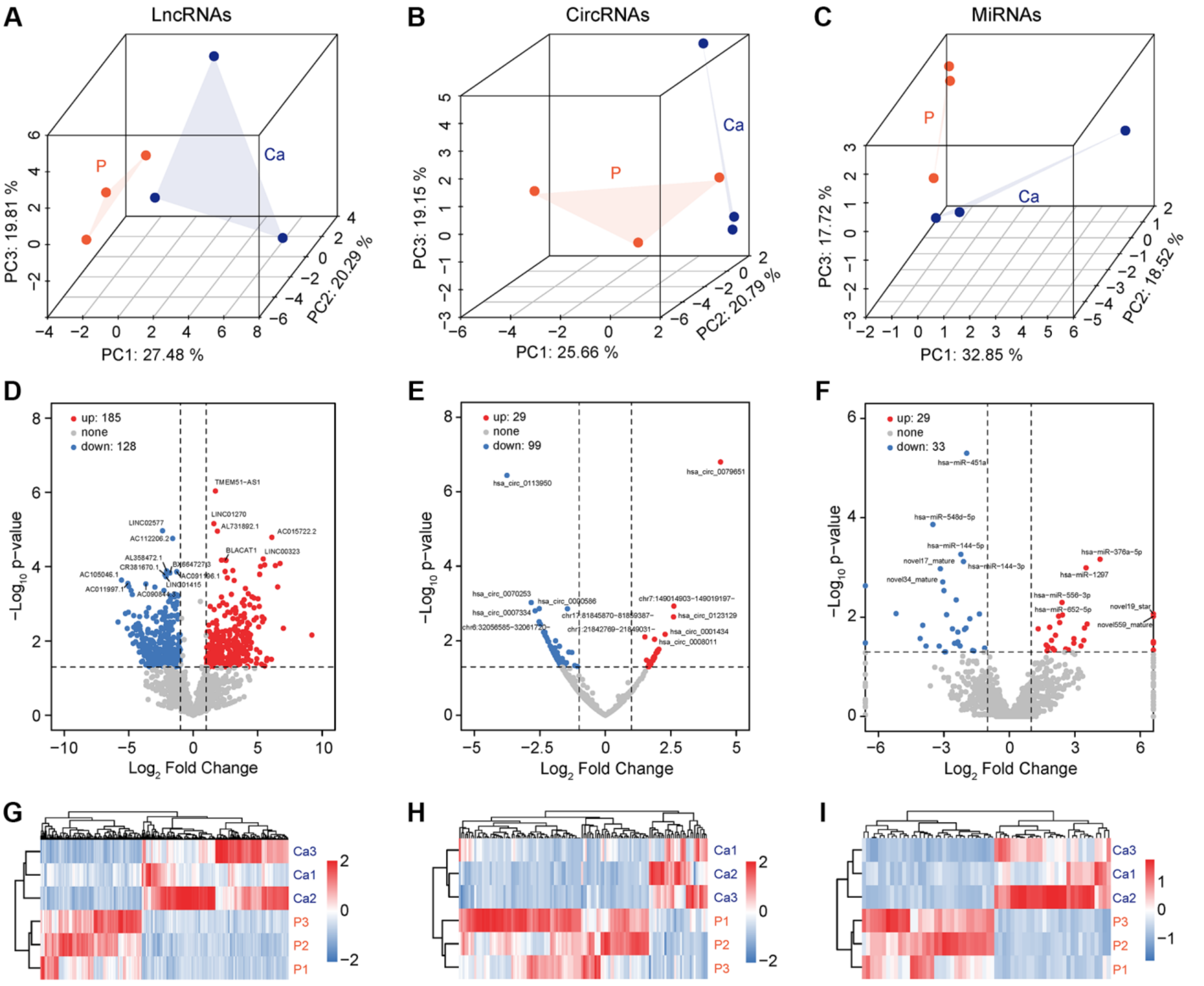

Supplementary Figure 6. Expression landscape of lncRNAs, circRNAs, and miRNAs in LUAD, related to Figure 3. (**A**–**C**) Principal component analysis of lncRNAs (**A**), circRNAs (**B**), and miRNAs expression profile (**C**) shows the similarity and reproducibility among different biological replicates. (**D**–**F**) Volcano plot shows differentially expressed lncRNAs (**D**), circRNAs (**E**), and miRNAs (**F**) between LUAD and non-tumor samples. (**G**–**I**) Heat map shows hierarchical clustering of differentially expressed lncRNAs (**G**), circRNAs (**H**), and miRNAs (**I**).

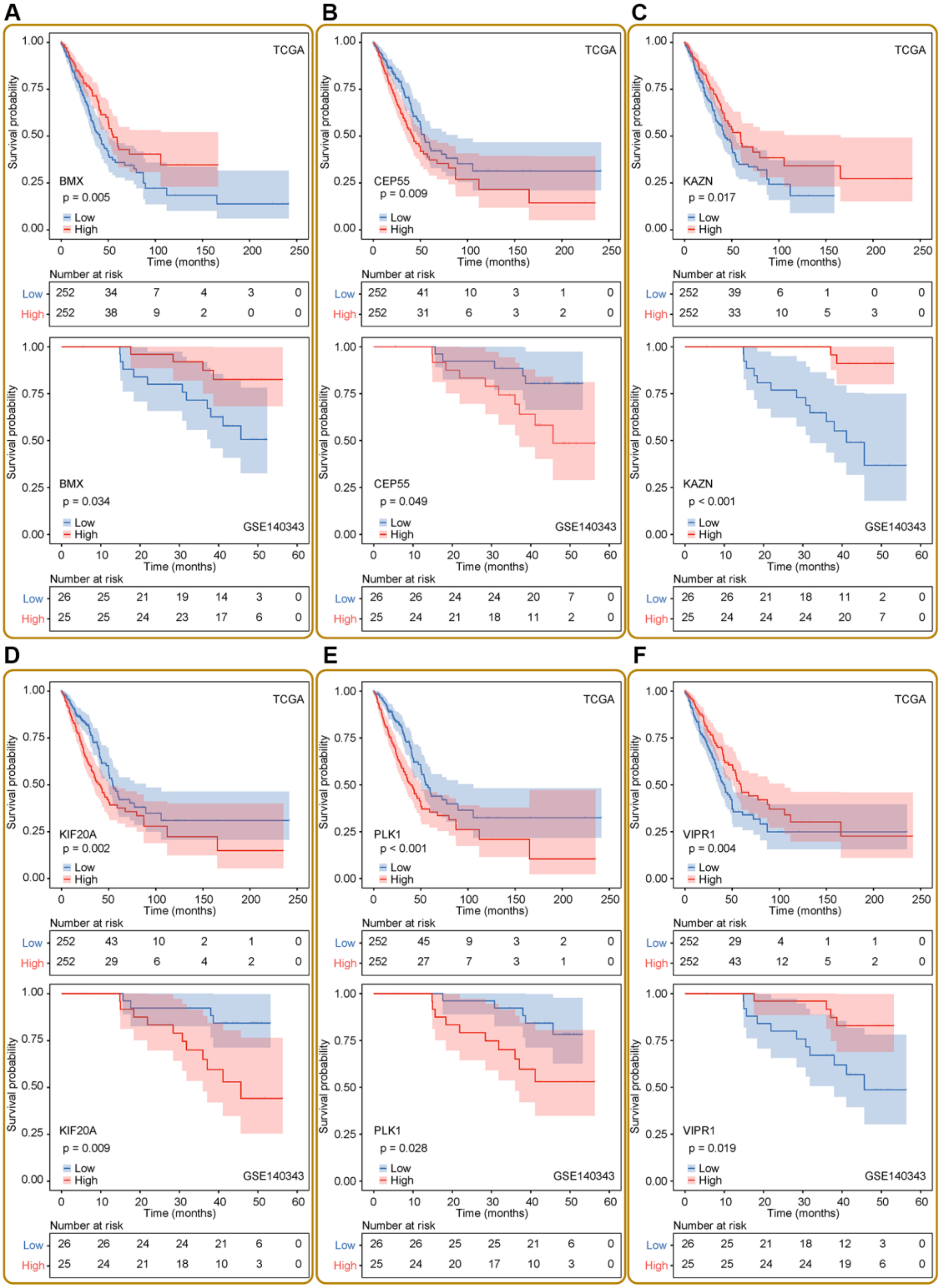

Supplementary Figure 7. The relationship between overall survival time of LUAD patients and the expression level of six prognostic factors in tumors according to TCGA and Chinese large-scale LUAD (GSE140343) transcriptome data, related to Figure 5. (**A**–**F**) Kaplan-Meier curves show BMX, CEP55, KAZN, KIF20A, PLK1, and VIPR1, respectively.

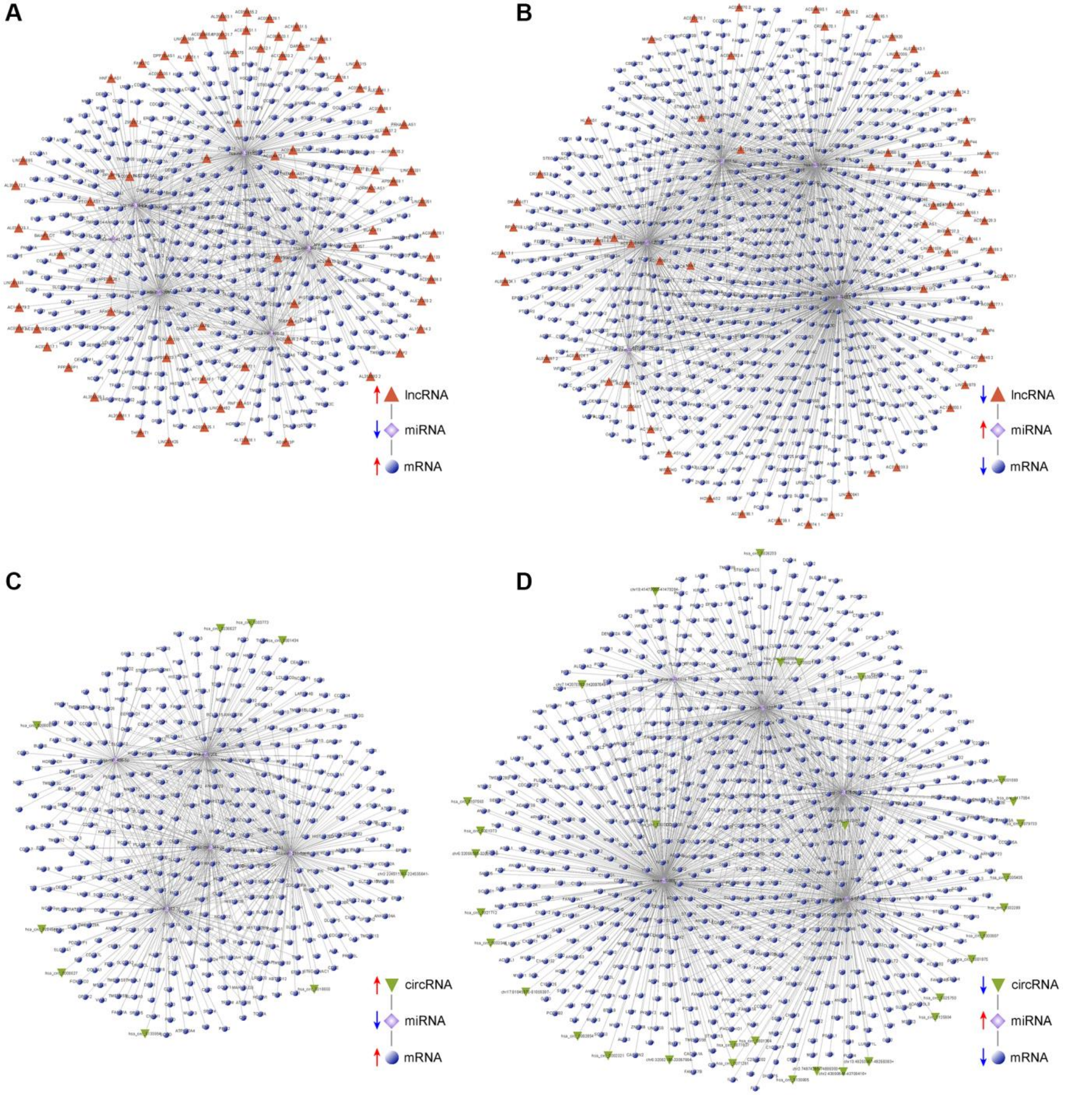

Supplementary Figure 8. Competing endogenous RNA (ceRNA) networks analysis based on differentially expressed coding genes and non-coding RNAs, related to Figure 3. The four networks represent upregulated lncRNAs (**A**), downregulated lncRNAs (**B**), upregulated circRNAs (**C**), and downregulated circRNAs (**D**), respectively.

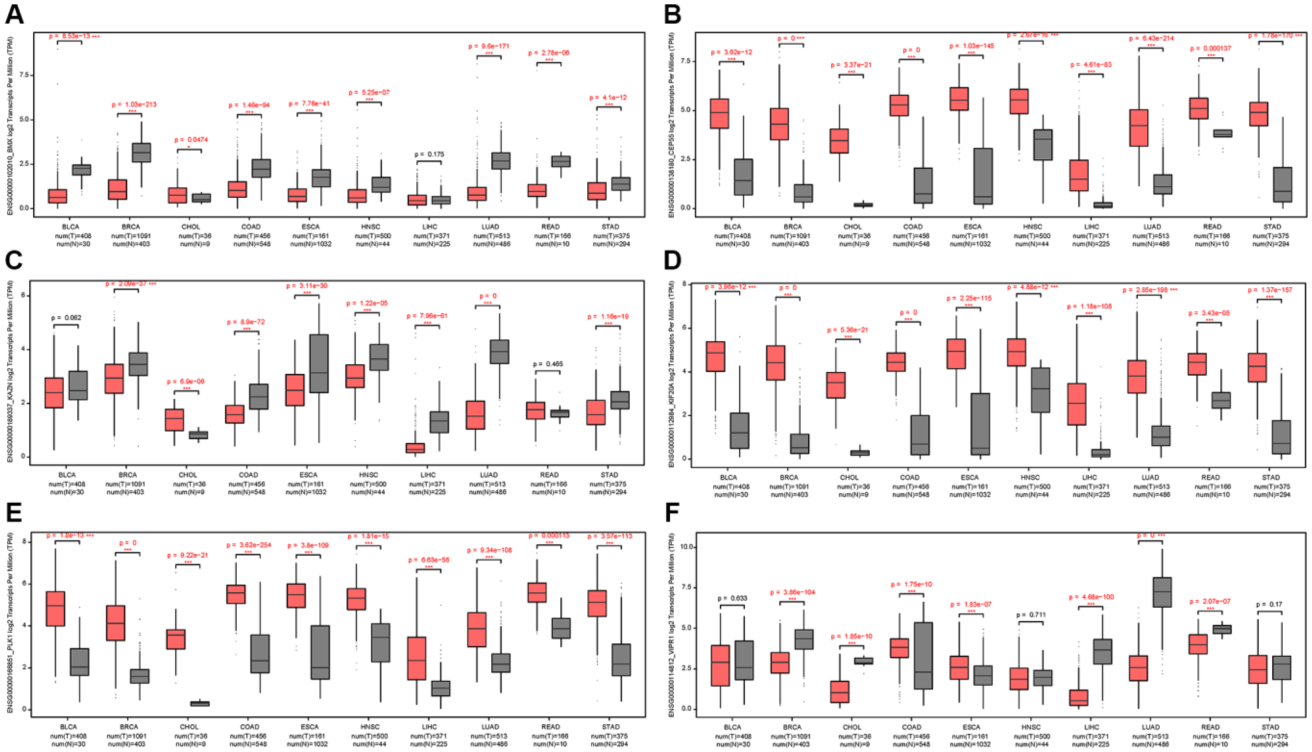

Supplementary Figure 9. The relative expression of six prognostic factors among different types of tumors according to TCGA transcriptome data, related to Figure 5. (**A**–**F**) Boxplot shows BMX, CEP55, KAZN, KIF20A, PLK1, and VIPR1, respectively.
