# Supplementary material for: Integrated multi-omics analysis for lung adenocarcinoma in Xuanwei, China
Source: Aging (Albany NY). 2023 Dec 13;15(23):14263–91. doi: 10.18632/aging.205300 (PMC10756121; doi:10.18632/aging.205300)
Supplement: Supplementary Table 1 [file aging-15-205300-s002.pdf]

SUPPLEMENTARY TABLE

Supplementary Table 1. Patient data of six samples.

Sample	Patient id	Type	Age	Sex	Smoker	Stage	TNM
Ca1	1	Tumor	57	Female	No	Ia	TisN0M0
P1	1	Non-tumor	57	Female	No	Ia	TisN0M0
Ca2	2	Tumor	65	Female	No	Ia	TisN0M0
P2	2	Non-tumor	65	Female	No	Ia	TisN0M0
Ca3	3	Tumor	56	Female	No	IIIb	T4N2M0
P3	3	Non-tumor	56	Female	No	IIIb	T4N2M0
