# Supplementary material for: Integrated multi-omics analysis for lung adenocarcinoma in Xuanwei, China
Source: Aging (Albany NY). 2023 Dec 13;15(23):14263–91. doi: 10.18632/aging.205300 (PMC10756121; doi:10.18632/aging.205300)
Supplement: Supplementary File 1 [file aging-15-205300-s007.pdf]

SUPPLEMENTARY FILE

Supplementary File 1. The main parameters used in the bioinformatical analysis.

Software	Version	Parameters
fastp	0.19.5	--length_required 50
RseQC	2.6.4	default
fastqc	v0.11.5	default
hisat2	2.2.1.0	--rna-strandness rf --fr
stringtie2	1.3.3b	--rf
bwa	0.7.5a	mem -M -R
samtools	1.3.1	mpileup -uRf -d 1000000
ASprofile	1.0.4	default
cpc2	beta	default
CNCI	1	-m
Pfam	v30	-e_seq 0.001
PLEK	1.2	default
CIRI	v2.0.3	default
miranda	v3.3a	-sc 150 -en -30 -strict
find_circ	v1.2	default
TargetFinder		default
bowtie2	2.2.9	-k30 -t
eXpress	1.5.1	--rf-stranded
DESeq2	1.18.0	q-value < 0.05, |log2FoldChange|>1
htseq-count	0.9.1	-s reverse
